# Circulating Tumor DNA—A Novel Biomarker of Tumor Progression and Its Favorable Detection Techniques

**DOI:** 10.3390/cancers14246025

**Published:** 2022-12-07

**Authors:** Xiaosha Wen, Huijie Pu, Quan Liu, Zifen Guo, Dixian Luo

**Affiliations:** 1 Hunan Province Cooperative Innovation Center for Molecular Target New Drug Study, Hunan Provincial Key Laboratory of Tumor Microenvironment Responsive Drug Research, School of Pharmaceutical Science, Hengyang Medical School, University of South China, Hengyang 421001, China; 2 Laboratory Medicine Centre, Huazhong University of Science and Technology Union Shenzhen Hospital, Shenzhen 518052, China

**Keywords:** cancer, ctDNA, detection technology, prognosis, medication guide

## Abstract

**Simple Summary:**

ctDNA is a small DNA fragment derived from tumor cells, which contains tumor-related genomic information, such as mutation, methylation, microsatellite instability, etc. It is an ideal biomarker for real-time monitoring of tumor development. This work mainly reviews the different sources of ctDNA, such as blood, urine, uncommon cerebrospinal fluid, ascites, etc. The most frequent mutation and methylation detection methods in ctDNA, such as the most commonly used high-throughput sequencing and innovative methods, combined with new materials in recent years, such as CRISPR-Cas system, graphene, etc. Finally, it is concluded that ctDNA has a comprehensive and accurate application value in all stages of tumor development (early screening, diagnosis, molecular typing, guiding medication, prognosis, and recurrence monitoring).

**Abstract:**

Cancer is the second leading cause of death in the world and seriously affects the quality of life of patients. The diagnostic techniques for tumors mainly include tumor biomarker detection, instrumental examination, and tissue biopsy. In recent years, liquid technology represented by circulating tumor DNA (ctDNA) has gradually replaced traditional technology with its advantages of being non-invasive and accurate, its high specificity, and its high sensitivity. ctDNA may carry throughout the circulatory system through tumor cell necrosis, apoptosis, circulating exosome secretion, etc., carrying the characteristic changes in tumors, such as mutation, methylation, microsatellite instability, gene rearrangement, etc. In this paper, ctDNA mutation and methylation, as the objects to describe the preparation process before ctDNA analysis, and the detection methods of two gene-level changes, including a series of enrichment detection techniques derived from PCR, sequencing-based detection techniques, and comprehensive detection techniques, are combined with new materials. In addition, the role of ctDNA in various stages of cancer development is summarized, such as early screening, diagnosis, molecular typing, prognosis prediction, recurrence monitoring, and drug guidance. In summary, ctDNA is an ideal biomarker involved in the whole process of tumor development.

## 1. Introduction

**Figure 1:**
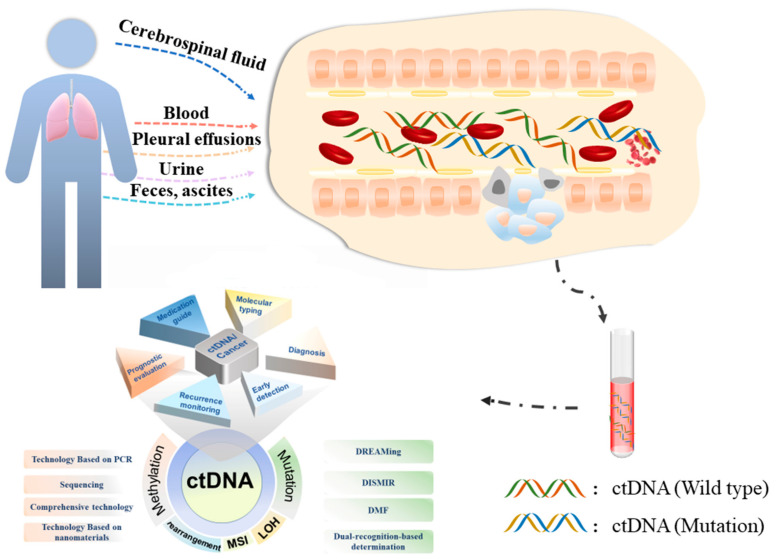
Overview of ctDNA analysis.

Cancer is a major public health concern worldwide and is the second leading cause of death after heart disease in the United States, resulting in 599,601 deaths in 2019 [1]. With an in-depth study of cancer, many researchers have proposed that cancer is an ecological disorder, not an isolated island that has nothing to do with the outside world. Inflammation and wound injury may promote the development of tumors by changing the tumor microenvironment, such as blood vessels, monocytes and macrophages, basement membranes, fibroblasts, and myofibroblasts [2]. An interesting study also proposed that human cancer is a multi-dimensional pathological ecosystem related to the unity of ecology and evolution, in which cancer cells cannot develop alone, but constantly interact and co-evolve with stromal cells in time and space, which is particularly evident in tumor metastasis. As per the ‘soil and seed’ theory, circulating tumor cells can be colonized in metastatic sites in a favorable environment, such as an acidic local environment produced by aerobic glycolysis and epithelial–mesenchymal transformation reprogramming induced by immunosuppression, promoting tumor inflammation, angiogenesis, etc. [3]. Hence, tumor treatment is not only limited to the tumor itself. Starting from the disordered ecological changes can come an innovative and effective treatment method, bringing unexpected therapeutic effects. Similarly, the detection of tumors should not be limited to the tumor itself. Detection of changes in the tumor microenvironment may also have unique detection values, such as epithelial–mesenchymal transition markers (E-cadherin, N-cadherin, and vimentin) (Figure 1).

From 1990 to 2019, the overall incidence rates and overall mortality rates of cancers have shown a steady downward trend owing to the development of early diagnosis, surgical techniques, and targeted therapy [1]. For example, serum prostate-specific antigen testing promoted the detection of latent asymptomatic diseases among men aged 65 years and older, reducing the mortality of patients with prostate cancer [4]. Cancer detection plays an extremely significant role in screening, intervention, and targeted therapy, thus, improving a patient’s quality of life. Due to the large investment of funds and continuous technological advances, a large number of early detection technologies have emerged in the field. There are several traditional detection technologies, such as classical biomarkers, imaging, and tissue biopsy. Biomarkers have been used in clinical settings with high sensitivity; however, their low specificity has always been an obstacle [5]. Moreover, the concentration of some biomarkers is so low that they cannot be detected by appropriate means [6]. A variety of imaging techniques have accelerated the diagnosis of cancer. To obtain more accurate results, different methods, such as pathological tests and magnetic resonance imaging (MRI), are usually combined, while the false positive rate and low efficiency are problems that need to be solved. For instance, the findings of mammograms are not obvious in patients with dense breasts, which is primarily attributed to low efficiency [7]. Tissue biopsies have always been the gold standard for cancer diagnosis, but its invasiveness limits its applications. Moreover, a tissue biopsy causes patient discomfort and cannot solve the problem of tumor heterogeneity due to its inability to examine the full range of tumor samples. The current detection technology achievements are gratifying. Cervical cancer detection technology (cytology, visual inspection with acetic acid, and molecular tests mainly for high-risk HPV DNA-based tests) and colorectal cancer screening technology (Guaiac-based Fecal Occult Blood Test, sigmoidoscopy) are widely used in clinical practice and have good screening effects [8,9]. However, breast cancer screening technology (mammography, ultrasound, and magnetic resonance imaging), prostate cancer marker detection (prostate-specific antigen, PSA), and hepatocellular carcinoma marker detection (alpha-fetoprotein, AFP) suffer from high false positive rates and limited sensitivity [10,11,12], and gastric cancer detection technology (gastroscopy) has poor patient compliance, which is not enough to solve clinical difficulties. Thus, more effective approaches should be explored in the coming years.

Recently, liquid biopsies have been favored by many researchers for their non-invasive, timely, and comprehensive characteristics. They are an emerging detection technology for cancer and contain circulating tumor cells (CTCs); circulating tumor DNA (ctDNA); circulating cell-free RNA (cfRNA), including small RNAs and mRNAs; circulating extracellular vehicles, including exosomes, proteins, and metabolites; tumor-educated platelets, which are almost always obtained from peripheral blood and other easily obtainable biological fluids, such as feces, urine, saliva, ascites, cerebrospinal fluid, and pleural effusions [13,14,15]. CTCs are a kind of tumor cell present in peripheral blood that is regarded as a marker related to tumor recurrence and prognosis. Chelain et al. reported that CTCs were detectable in 399 of 1697 patients with breast cancer in the NCDB cohort (23.5%) and in 294 of 1681 patients in the SUCCESS cohort (19.4%). CTC-positive, early-stage patients who were treated with radiotherapy after breast-conserving surgery in the NCDB and SUCCESS cohorts showed longer local recurrence-free survival, disease-free survival, and overall survival. However, for patients with CTC-negative or CTC-positive breast cancer, the overall survival was not related to radiotherapy after mastectomy [16]. Moreover, the CTC count was found to be an accurate method to support prognostic information. In castration-resistant prostate cancer, the CTC dynamics from 5–50 during therapy revealed an improved overall survival and were evaluated as an intermediate endpoint of the clinical outcome [17]. The reason why CTCs originating from tumors play a considerable role in cancer metastasis is that CTCs circulate in lymphatic and blood vessels, spread, and implant in distant organs through epithelial–mesenchymal transition. Further, the metastatic efficiency of CTC clusters is 23–50 times higher than that of a single CTC [18,19]. Li and his partners [20] developed cancer membrane-coated digoxin (DIG) and doxorubicin (DOX) co-encapsulated PLGA nanoparticles (CPDDs) to significantly target and precisely disaggregate CTC clusters. Moreover, CPDDs could inhibit the process of epithelial–mesenchymal transition, thus accomplishing an efficient anti-metastasis clinical outcome. cfRNA is also a valuable cancer marker. Matthew et al. [21] screened out tumor-specific cfRNA biomarkers from the plasma of individuals with and without cancer, called dark channel biomarker (DCB) genes. There were DCBs specific for lung cancer, such as *SLC34A2*, *GABRG1*, *ROS1*, *AGR2*, *GNAT3*, *SFTPA2*, *MUC5B*, *SFTA3*, *SMIM22*, *CXCL17*, *BPIFA1*, and *WFDC2*, as well as for breast cancer, such as *CSN1S1*, *FABP7*, *OPN1SW*, *SCGB2A2*, *LALBA*, *CASP14*, *KLK5*, and *WFDC2*. Unexpectedly, there were a few differentially expressed DCB genes in certain cancer subtypes. The DCB gene *FABP7* was downregulated in the cfRNA of patients with hormone receptor-positive breast cancer and upregulated in patients with triple-negative breast cancer. Moreover, the ability to detect DCB genes was related to the tumor fraction of the plasma, suggesting that cfRNA could be a detectable biomarker for a variety of cancers and cancer subtypes. However, the strategies for the accurate capture of real CTCs in a complex blood environment and the degradation of RNA are major obstacles in clinical applications and pose great challenges to the effectiveness and accuracy of detection results.

Importantly, ctDNA is a cornerstone in liquid biopsies. ctDNA is secreted into the bloodstream through apoptosis, necrosis, and the active release of tumor cells. Thus, it reflects related alterations of the tumor characteristics, such as genetic mutations, gene rearrangements, epigenetic changes, microsatellite instability (MSI), and loss of heterozygosity [22,23]. Moreover, ctDNA has a short half-life of about 2 h and consists of 70–200 base pairs. Thus, ctDNA has been able to reflect the progression of cancer in a timely manner. An overwhelming number of researchers have studied how ctDNA plays a valuable role in cancer diagnosis, treatment, monitoring, prognosis, and relapse evaluation. For instance, Gillian et al. [24] demonstrated a large number of somatic alterations in blood samples and same-patient tumor tissue samples from 104 patients with metastatic urothelial carcinoma and found that aggressive disease could be predicted by high ctDNA levels. Meanwhile, as for real-time genomic biomarker assessment, ctDNA was more beneficial than tumor tissue. In the I-SPY 2 TRIAL, where 84 patients with early breast cancer were treated with standard neoadjuvant chemotherapy alone or in combination with an AKT inhibitor, Magbanua et al. [25] reported that ctDNA was detected before, during, and after neoadjuvant chemotherapy treatment, and it was a significant prognostic factor for patient survival analysis after neoadjuvant chemotherapy treatment. In gastric cancer, cancer progression has been evaluated by genomic alternations in ctDNA earlier than with CT scanning, a standard evaluation method for the therapeutic response. Moreover, novel genomic changes in ctDNA have the potential to reflect resistance to treatment with Pyrotinib, a dual EGFR/HER2 tyrosine kinase inhibitor [26]. Altogether, ctDNA is an ideal biomarker for cancer, and it is worthwhile to analyze and summarize ctDNA-related processes.

In this review, we will discuss: (1) the sources of ctDNA, ctDNA preparation, and individualized technologies for sample acquisition; (2) detection technologies for ctDNA; (3) the combination of ctDNA and treatment strategies.

## 2. Biological Characteristics of ctDNA

In 1948, a fraction-free DNA fragment, cfDNA, was found in human blood plasma [27]. ctDNA is a type of cfDNA associated with the development of a tumor. However, where the source of ctDNA is not specified concretely, ctDNA mainly originates from programmed apoptosis or necrosis of tumor cells through a series of source comparison results [28]. This concept has always been embedded in the minds of researchers, but Allenson et al. [29] found that circulating exosomes in blood samples from cancer patients also contain the same mutation sites as ctDNA, such as the *KRAS* gene G12A. Moreover, the proportion of gene mutations in circulating exosomes is higher than that in ctDNA in the blood, indicating that circulating exosomes containing multiple components may be another source of ctDNA, broadening the origin of ctDNA, and providing new ideas for accurate ctDNA analysis. A deeper understanding of the source of ctDNA can further clarify the changes at specific genetic levels in various types of cancer, which is conducive to subsequent targeted drug research and therapy. The ctDNA reveals tumor-associated DNA changes at the genetic level, including gene mutations, DNA methylation, MSI, gene rearrangements, and loss of heterozygosity. Microsatellite instability refers to the phenomenon that a microsatellite locus in a tumor has a new microsatellite allele due to the insertion or deletion of repeat units compared with normal tissues [30]. The occurrence of MSI is due to functional defects in the DNA mismatch repair in tumor tissues, and MSI with DNA mismatch repair deficiency is an important clinical tumor marker. Markus et al. [31] first detected MSI in cfDNA and ctDNA from patients with prostate cancer, and the results were consistent with the results of tissue whole-exome sequencing in MSI-positive patients with metastases. Moreover, a sharp decrease in ctDNA levels was observed in three patients with mismatch repair deficiency/high MSI metastatic colorectal cancer after the administration of nivolumab, a cytotoxic T lymphocyte-associated protein-4 (CTLA-4) inhibitor, and pembrolizumab, an FDA-approved first-line drug for dMMR/MSI-H metastatic colorectal cancer [32], indicating that MSI in ctDNA is still a clinical biomarker. In addition, Han et al. developed an MSI bioinformatics tool based on cfDNA sequencing data with a sensitivity of 100% and a detection limit of 0.05% ctDNA content [33]. The loss of heterozygosity means that, in a pair of alleles at a specific locus on a homologous chromosome, one side has a harmful mutation, whereas the other side is normal, resulting in a semi-homozygous or homozygous gene locus [34]. The loss of heterozygosity is the major form of mutation in the *NF1* gene. A previous study showed that 11 out of 14 patients with invasive lobular or ductal breast carcinoma have *NF1* gene loss of heterozygosity, and this alternation was associated with endocrine therapy resistance and activation of the RAS/RAF kinase, indicating that patients’ prognoses will be better after they are treated with an appropriate kinase inhibitor [35]. A gene rearrangement, a common form of a genetic level change, is the repair result of an intragenic or intergenic rearrangement of DNA after double-strand breaks, including a gene fusion. A *CAD-ALK* gene fusion was found in the second-generation sequencing spectra of ctDNA derived from the blood and urine of patients with metastatic colorectal cancer. The combined rearrangement of *CAD* gene’s exon 35 and *ALK* gene’s exon 20, as well as the dynamic changes in the *CAD-ALK* gene fusion, were consistent with clinical progression in patients [36].

Gene mutations are referred to as changes in the base pair composition or arrangement order in a gene structure, including point mutations, frameshift mutations, deletion mutations, and insertion mutations. DNA methylation is an epigenetic modification, which is catalyzed by DNA methyltransferase (DNMT). S-adenosylmethionine (SAM) serves as a methyl donor, and methyl is added selectively to the DNA of two CG nucleotides of cytosine. Mainly, 5-methyl cytosine (5-mC) is formed, which is common in the gene 5′-CG-3′ sequence. DNA methylation could lead to changes in the chromatin structure of the corresponding regions of the genome, causing DNA to lose the cutting site of the ribozyme or restriction endonuclease and the sensitive site of the DNA enzyme. This leads to the formation of chromatin that is highly helical and condensed into clusters, which loses its transcriptional activity [37].

ctDNA, which is derived from tumor cells, contains tumor-specific gene mutation sites and methylation sites, such as the EGFR gene in non-small cell lung cancer (NSCLC), a *BRCA1/2* mutation and methylation in breast cancer, a *KRAS* gene mutation in colorectal cancer, and a *BRAF* gene mutation in thyroid cancer. In addition, in the process of ctDNA detection, a mutation or methylation can be identified and determined by sequencing results or corresponding sensing signals, including current changes, optical changes, and absorbance. Gene mutations and DNA methylation are the two main aspects which have been summarized below.

## 3. ctDNA Preparation

The process of ctDNA detection includes ctDNA preparation, the establishment of a related library, analysis, and data integration and comparison. During this process, ctDNA preparation is the first step and determinant for improved results. First, the origin of ctDNA is a key factor and is diverse, such as blood, urine, feces, saliva, ascites, cerebrospinal fluid, and pleural effusions. Second, the selection of methods to extract ctDNA is also vital for different sources. The preparation stage is mainly divided into two steps: sample processing and ctDNA extraction.

### 3.1. Blood

Blood is the most common origin for ctDNA extraction in almost all solid tumors, such as esophageal cancer [38,39], NSCLC [40], colorectal cancer [41], hepatocellular carcinoma [42], and early-stage breast cancer [43]. First, blood is drawn from the examined subjects and collected in EDTA blood collection tubes, if the samples will be processed within 2 h, or in preservative tubes, if the samples will be processed within 46–72 h after the venipuncture. Interestingly, the detection efficiency of different sources of ctDNA, such as plasma, serum, and peripheral blood, is different. The detection efficiency of plasma was found to be better than that of other sources, and the detection specificity and sensitivity were 0.96 and 0.78, respectively [44]. Further, the collection efficiency is impacted by the choice of tubes, and appropriate tubes can expand the scope of blood collection in the clinic. For example, EDTA, CellSave tubes, and Streck BCT had a similar ability to preserve blood after 6 h of blood sample collection, while CellSave tubes and Streck BCT could stabilize wild-type DNA and ctDNA at 48 h [45]. The specimen is then centrifuged at 1600× *g* for 10 min, and the supernatant is collected and centrifuged at 16,000× *g* for 10 min. Second, the extraction kit is used to precisely obtain ctDNA, and there are currently many commercially available ctDNA extraction kits for clinical and scientific research, such as QIAsymphony DSP Virus/Pathogen Midi Kit (Qiagen, Hilden, Germany), QIAsymphony DSP Circulating DNA Kit (Qiagen, Hilden, Germany), MagMAX™ Cell-Free DNA Isolation Kit (Thermo Scientific, Waltham, MA, USA), QIAamp Circulating Nucleic Acid Kit (Qiagen, Hilden, Germany), and EliteHealth cfDNA extraction Kit (EliteHealth, Pembroke Pines, FL, USA). Several extraction kits are presented in Table 1.

### 3.2. Urine

Urine is a more convenient source of ctDNA than blood, especially for renal cancer [46], colorectal cancer [47], bladder cancer [48], and urothelial cancer [49], because sample collection is performed without discomfort to the patient. Urine is divided into a urine supernatant (USN) and a urine cell pellet (UCP) for ctDNA collection. Generally, 30–50 mL of urine is collected from patients in 50 mL Falcon tubes, and 0.5 M EDTA is added to the Falcon tubes within an hour of collection. After EDTA dissolution, the specimen is centrifuged at 2400× *g* for 10 min. Then, separate cryotubes are used for the supernatant and are stored at –80 °C. If one wants to obtain a UCP collection, the above 1 mL supernatant is added to the primary Falcon tube containing UCP, and the suspension is transferred into a 2 mL sterile microfuge tube after gentle agitation. The sediment is staged at –80 °C through a spun rate at 13,300 rpm for 10 min. Moreover, the QIAamp Circulating Nucleic Acid Kit (Qiagen, Hilden, Germany) could also be used for ctDNA purification from urine.

### 3.3. Cerebrospinal Fluid

Central nervous system (CNS) diseases account for a large proportion of disorders that particularly affect the quality of life of patients. Blood-derived ctDNA is gradually becoming a novel biomarker for cancer, but the concentration of ctDNA in the cerebrospinal fluid is higher than that in the plasma of patients with brain tumors [50,51]. Taken together, cerebrospinal fluid is more closely related to the origin of CNS tumors than plasma [52,53]. Cerebrospinal fluid samples are often obtained from lumbar punctures of patients, and after centrifugation, the QIAsymphony DSP Virus/Pathogen Midi Kit (Qiagen, Hilden, Germany), the QIAsymphony DSP Circulating DNA Kit (Qiagen, Hilden, Germany), and the QIAamp Circulating Nucleic Acid Kit (Qiagen, Hilden, Germany) can be used for ctDNA extraction.

### 3.4. Feces, Ascites, and Pleural Effusions

Feces, ascites, and pleural effusions are supplemental sources for ctDNA extraction. Through relative centrifugation, the supernatant is collected with a Fast DNA Stool Mini Kit (Qiagen, Hilden, Germany) [54] and QIAamp Circulating Nucleic Acid Kit (Qiagen, Hilden, Germany) [55]. All selective kits for ctDNA extraction are shown in Table 1.

## 4. Detection Technology of ctDNA

Gene mutations and DNA methylation are two major components of ctDNA detection. Changes in the driving genes of tumors could often break the dynamic balance between proto-oncogenes and tumor suppressor genes and promote the expression of oncogenes, thus causing tumors. Moreover, the change in the driving gene is often a single base change, which is a great difficulty for the detection of ctDNA with extremely low content. DNA methylation is also key to ctDNA detection. The excess methyl group on the cytosine in the driving gene could hinder transcription by affecting the binding of transcription factors to their target fragments. Therefore, DNA methylation plays the same role as DNA mutations in terms of causing tumorigenesis. In this regard, researchers have developed many effective detection methods, which will be reviewed below.

### 4.1. Mutation Detection of ctDNA

#### 4.1.1. Based on PCR

Based on the principle of PCR, a series of PCR-related methods have been developed that have also been applied in ctDNA detection. Such methods include amplification refractory mutation system-PCR (ARMS-PCR); co-amplification at lower denaturation temperature-PCR; (COLD-PCR); droplet digital PCR (ddPCR); beads, emulsions, amplification, and magnetics (BEAMing); qPCR.

(1) ARMS-PCR

ARMS-PCR is also known as allele-specific PCR. Its detection principle is to control the allele-specific extension by a 3’ end primer design and to detect the fluorescence signal value in combination with a TaqMan probe method, so as to distinguish between wild-type alleles and mutant genes. Maryam et al. [56] used the tetra-primer amplification refractory mutation system-polymerase chain reaction (T-ARMS-PCR) method to accurately detect the fat mass and obesity-associated gene (FTO) polymorphism (rs9939609) in the blood samples from patients with colorectal cancer. It was found that disease progression in patients with colorectal cancer was positively correlated with the A allele of the rs9939609 polymorphism, but the underlying mechanism needs further exploration. Similarly, the ARMS-PCR method was used to detect the mutation rate of ESR1 in 43 patients with metastatic breast cancer treated with aromatase inhibitors, and the mutation rate was 27.9% (12/43). To prove the accuracy of this method, the detection rates of ESR1 between ARMS-PCR and absolute quantitative ddPCR were also compared, and the detection consistency of the two methods was found to be 95.45% (*p* < 0.001) [57]. In general, ARMS has a high sensitivity and accuracy, convenient operation, and low cost. However, due to the need for primer design for mutation targets in this method, a large number of screenings are needed for primer optimization in the early stages. Compared with Sanger sequencing and next-generation sequencing, it is impossible to achieve high-throughput and high-position detection [58].

(2) COLD-PCR

COLD-PCR is a low-temperature PCR developed in recent years, which solves the shortcomings of low sensitivity and low specificity in traditional PCRs. In the process of DNA double-strand synthesis, the alternation of single nucleotides makes the denaturation temperature of the sequence produce small and predictable changes. In COLD-PCR, in order to generate molecular hybridization between mutant genes and wild-type alleles, an annealing temperature should be set in the PCR cycle. The annealing temperature of heterotypic double-strand hybridization is lower than that of homotypic double-strand hybridization. Therefore, heterotypic double-strand hybridization has degenerated at a lower annealing temperature during amplification when the homotypic double-strand is still in the state of double-strand, which cannot be efficiently amplified. By setting the denaturation temperature at a lower annealing temperature, COLD-PCR can amplify a large number of mutants anywhere, while the wild-type content remains unchanged. Thus, the goal of enriching mutant genes is achieved, which can be used to detect low-abundance DNA. Jensen et al. [59] detected a significant *BRAF* V600E mutation in the plasma (*cfBRAF* V600E) of patients with papillary thyroid cancer by using the combined method of COLD-PCR and microfluidic digital PCR; the sensitivity of the combined method increased more than 100-fold in comparison to single digital PCR. Moreover, cfBRAF V600E was correlated with the tumor size, pulmonary micro-metastases, and extrathyroidal gross extension, indicating that it might be an independent factor for the progression of papillary thyroid cancer. Before absolute quantification using second-generation sequencing or digital PCR, enhanced-ice-COLD-PCR was carried out to enrich the template, and the enrichment efficiency was 100-fold higher compared to digital PCR, which greatly improved the detection rate of the *ESR1* gene mutation in blood samples from patients with metastatic estrogen-positive breast cancer [60]. Silvia et al. compared the detection rates of gene mutations in patients with metastatic colorectal cancer through COLD-PCR, a microarray, and ddPCR. In terms of blood samples, the detection coincidence rate of COLD-PCR was the highest, reaching 92.6% [61]. In summary, COLD-PCR has a strong advantage in the detection of rare and low-content mutations, but its main function is to enrich the detection template. It needs to be combined with other detection methods, such as ddPCR and next-generation sequencing, to play a greater role in ctDNA detection.

(3) BEAMing

BEAMing, proposed by Devin et al. [62] is a method for the measurement and quantification of a single DNA molecule with high reliability and sensitivity. The main process includes the following steps: a specific combination of primers and magnetic beads, formation of micro-emulsions in the oil phase by the DNA template and probes of coating primers, a PCR in the oil phase, purification of the magnetic beads after PCR by magnetism, identification of different DNA molecules, and, finally, detection of DNA by various methods, including flow cytometry, fluorescence microscopy, and LSR I and II. The specific experimental steps have been described by Ococks et al. [63].

BEAMing has become a tool that many researchers choose to detect ctDNA due to its high sensitivity. Kagawa et al. used BEAMing technology to verify the consistency of a *RAS* gene mutation in 216 patients with colorectal cancer with a single site metastasis and in liquid biopsy. The consistency was up to 91% for liver metastases without considering the influence of factors such as the maximum diameter of the tumor and the number of lesions, suggesting that gene mutational analysis in ctDNA may be used to predict the site of tumor metastases [64]. BEAMing was also used in the detection of a *ctIDH1* mutation in patients with glioblastoma with 100% specificity. This mutation indicated patients with a better survival prognosis [65]. Krug et al. combined exosomal RNA and ctDNA using a targeted, next-generation sequencing panel and BEAMing to improve the sensitivity of detecting *EGFR* mutations, thereby creating a reasonable therapeutic management strategy [66].

(4) ddPCR

ddPCR, also known as third-generation PCR technology, is a method for the absolute quantification of DNA with a 0.01% limit of detection compared to ARSM (0.1%) and Sanger (10%) sequencing. One of the characteristics of this method is that the PCR is carried out in water-in-oil droplets, and the fluorescence signal is detected in the reader. After analysis, absolute quantification of DNA is achieved without the housekeeping gene as the internal reference. It is widely used in the determination of viral load and early tumor screening due to its high sensitivity and still plays an important role in the detection of low content ctDNA mutations. In a meta-analysis comparing ddPCR and ARMS for detecting *EGFR* mutations in ctDNA [67], ddPCR showed greater sensitivity than ARMS, especially during the early stage of tumorigenesis. An optimized ddPCR, library aliquot-based ddPCR (LAB-ddPCR), successfully detected 41 T790M mutation cases in 70 patients with NSCLC who were treated with tyrosine kinase inhibitors, whereas conventional ddPCR detected only 27 cases [68].

**Figure 2:**
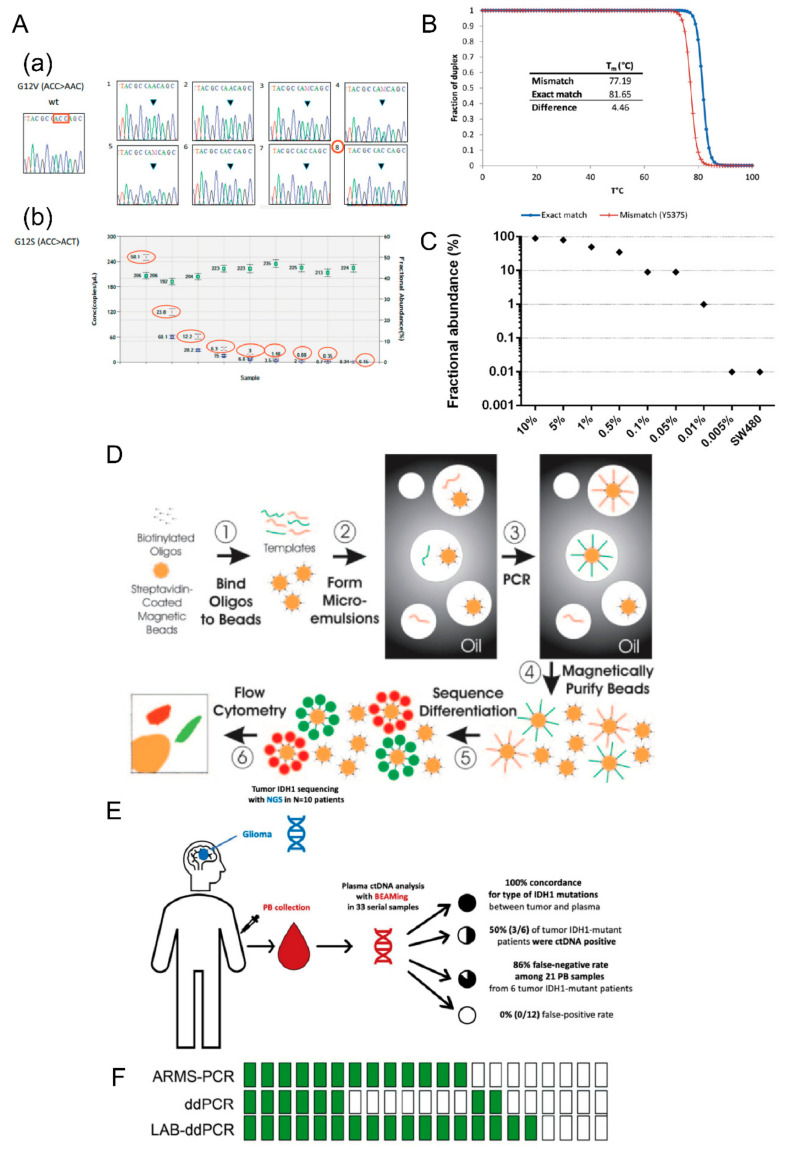
Detection technology of ctDNA mutation. (**A**) Sensitivity of COLD-PCR (**a**) and ddPCR (**b**) for G12V-mutated DNA of metastatic colorectal cancer patients [59]. Aa, fast COLD-PCR sequence profiles of G12 V mutated DNA serially diluted with wild-type DNA (1 = 12.5%, 2 = 6.25%, 3 = 3.12%, 4 = 1.56%, 5 = 0.78%, 6 = 0.39%, 7 = 0.2%, 8 = 0.1% of mutated DNA). The antisense sequence is shown. Ab, sensitivity of the ddPCR G12S assay in discriminating different proportions of mutated alleles on serial dilutions starting from 50% up to 0.1% of the mutated allele. The respective percentages of fractional abundance obtained for each point are circled in red. (**B**) Melting curves and melting temperatures (Tm) of the wild-type ESR1 and ESR1 Y537S mutation [61]. (**C**) Sensitivity of enhanced-ice-COLD-PCR assay for ESR1 [60]. (**D**) Schedule of BEAMing [63]. (**E**) Comparison of BEAMing and NGS in detecting IDH mutation in glioma patients [65]. (**F**) The sensitivity of LAB-ddPCR for the detection of ctDNA T790M mutation was further improved compared to ddPCR and ARSM-PCR [68].

However, a serious drawback is that the method requires a large peripheral blood volume (10 mL) when used for ctDNA, and the experimental conditions and reagents need to be optimized to solve this obstacle (Figure 2).

#### 4.1.2. Sequencing-Based Detection

With the progress of computer science and bioinformatics, sequencing has been gradually applied in the field of molecular biology to verify DNA sequences. Moreover, the breadth and depth of sequencing technologies have gradually been improving, from Sanger sequencing to next-generation sequencing and to single-molecule sequencing. For low ctDNA content, sequencing is an accurate detection method.

Sanger sequencing was developed by Frederick Sanger based on a chain-terminating inhibitor in 1977 [69]. There are many advantages to Sanger sequencing, such as a high resolution, long sequencing fragments, a detailed process, visual results, and low false detection rates. However, low flux is a fatal flaw and a major constraint in ctDNA detection.

Next-generation sequencing, also known as high-throughput sequencing, is currently the most common sequencing method after the continuous development and improvement of data technology, marked by Solexa Synthesis sequencing (Illumina), 454 Pyrosequencing (Roche), SOLiD Linkage Sequencing (ABI), Nanosphere Sequencing (BGI), and the Ion Torrent technology (Thermo Fisher). Its main steps include sample preparation, library construction, PCR amplification, sequencing, and data analysis. Samantha and Michael [70] summarized the concepts and methods of next-generation sequencing in ctDNA detection. They have not been summarized here.

#### 4.1.3. Comprehensive Technology

Enzymes are bioactive substances with high efficiency and affinity. One class of enzymes, such as the traditional restriction enzymes, could cut DNA or RNA. Once the DNA sequence contains the restriction enzyme corresponding to the cleavage site, it will be precisely cut. In recent years, with the rapid development of gene editing technology, a non-traditional restriction enzyme could also be used to efficiently cut the target fragment. To achieve the goal of detecting very few ctDNA mutants in the context of abundant wild-type molecules, the enzyme specifically cuts a large number of wild fragments to increase the percentage of mutant content. Then, it increases the net content of mutant fragments through various amplification techniques. Combined with sequencing and other signal detection methods, the mutation of the target gene in ctDNA could be detected successfully. Next, we will introduce several enzyme-based ctDNA mutation detection methods.

(1) CRISPR/Cas9 + PCR

The CRISPR-Cas system, known as a “gene editing scissor,” is a protective mechanism found in archaea, which specifically targets and cuts exogenous sequences. The most commonly used systems are Cas9, Cas12, and Cas13. Their function of “gene scissors” is not universal but is restricted by the PAM site, and their shear targets are different. Cas9 is used to cut double-stranded DNA, and the corresponding PAM site is 5 ‘-NGG-3’. Double-stranded DNA or single-stranded RNA could be cut by Cas12, and the corresponding PAM is 5’ -TTN/TTTN-3’. In particular, Cas13 is suitable for cutting RNA and is still limited by the protospacer flanking sequence rather than by the PAM site. It is worth mentioning that after the target fragments are specifically cut by Cas12 and Cas13, the trans-cleavage is activated, and the T-rich sequences could be cut non-specifically.

Based on the characteristics of the CRISPR-Cas system that specifically cuts the target fragment, Wang et al. [71] combined CRISPR-Cas9 with traditional PCR to achieve the detection of the most common deletion mutation in exon 19 of EGFR in NSCLC. A target single-stranded guide RNA (sgRNA) was designed for the deletion fragment, and Cas9 was combined with the target sequence under the guidance of sgRNA to achieve specific cutting of the wild sequence. The mutation sequence was not affected and was continuously enriched in the PCR amplification process. The identification of the mutation sequence was realized with Sanger sequencing with a 0.01% limit of detection. Although the sensitivity is high, the operation is extremely complex. The enzyme activity temperature of Cas9 is 37 °C, and it can, therefore, be easily inactivated in the PCR process. Therefore, Cas9 needs to be continuously added in the reaction process to maintain its enzyme digestion function. Based on this principle, Lee et al. detected KRAS mutations in ctDNA [72]. Moreover, Chen. et al. [73] proposed a method that combines Cas9 with graphene to detect an EGFR mutation. Through the reduction of L-ascorbic acid, Au–Pd–Pt nanoflower-decorated, three-dimensional (3D) graphene was formed by doping gold (Au), pterion (Pd), and platinum (Pt) into 3D graphene materials, which were immobilized in a glassy carbon electrode. The graphene detection platform was equipped with a capture probe, referred to as an entropy-driven strand displacement reaction (ESDR). Under the action of Cas9 and target sgRNAs, the mutant template could be cut to provide target primers for the amplification cycle. In the amplification cycle, a T1 fragment complementary to the capture probe can be generated, and the detection signal could be realized by the current in the electric field. This method abandons the conventional PCR enrichment process, saves detection time and processes, and has high sensitivity with a 0.13 pM detection limit. Meanwhile, the CRISPR-Cas9 cleavage-triggered ESDR nanoflower biosensor is applicable in clinical samples with high accuracy.

There is an inactive Cas9 protein, named deactivated Cas9 (dCas9), which does not interfere with the recognition of target fragments by sgRNA, but cannot specifically cut the target fragments. The combination of a graphene oxide screen printed electrode (GPHOXE) and dCas9 proteins and sgRNA was used to detect a PIK3CA E542K mutation in the ctDNA of patients with breast cancer. The GPHOXE is an easy-to-fix nanomaterial. The biorecognition complex composed of dCas9 and sgRNA can covalently bind to the oxidized carbonyl groups on the GPHOXE. After the target fragment is specifically recognized by the biorecognition complex, the resistance increases. The change in resistance can be analyzed by electrochemical impedance spectroscopy (EIS) to realize the detection of mutations in ctDNA. The CRISPR-dCas9-powdered impedimetric system had a short detection time of 40 s, and the detection limit was 1.92 nM. It had a linear relationship within the concentration range of 10–220 nM and showed high selectivity and high repeatability in clinical samples [74].

CRISPR-Cas12a was also applied to the BRAF V600E mutation in a recent publication [75]. A fluorescence detection strategy based on the three-dimensional DNA walker (3DDW) and cis- and trans-cleavage characteristics of CRISPR-Cas12a has been proposed, with DW–S–MB complex trajectories of hairpin DNA walker tracks (DWs). Substrate strands (SSs) and magnetic beads (MBs) are essential for the 3DDW, and DWs and SSs modified by biotins are ligated to MBs. There are complementary base sequences in DWs and between DWs and SSs. Further, DWs have a 7 nt ctDNA recognition sequence, and SSs have enzyme sites of the endonuclease Nb.BbvCI. Target sequences bind to their complementary gap in DWs as target ctDNA, and 3′ recognition sequences of DWs are exposed to connect to the complementary sequence of SSs. The hybridization structure is constructed by DWs, and SSs could be cut by Nb.BbvCI to release DWs and output DNAs (ODs). The released DWs are continuously hybridized with SSs, so that ODs are also constantly accumulated. After MDs are removed by magnetic separation, the supernatant is added to the CRISPR-Cas12a detection system, including Cas12a, a reporter modified by a fluorescent group, and a quenching group. ODs are cis-cleaved by Cas12a, and cuts in the reporter probe are trans-cleaved, thus releasing the fluorescence signal. A fluorescence spectrophotometer is used for analysis, and finally, the ctDNA detection purpose is achieved. The strategy shows a good linear relationship in the concentration range of 1 fM to 20 nM; the detection limit is 0.37 fM; the technique has high sensitivity and specificity. In the mixed ratio of mutant DNA and wild-type DNA, the strategy could distinguish the ratio of 1:100,000, and when different concentrations of mutant DNA were added to the serum samples of healthy volunteers, the method also exhibited a good detection effect, showing a good clinical application potential.

(2) Based on Functional enzymes

In addition to restriction endonucleases, ribonuclease HII (RNase HII) and deoxynucleotidyl transferase (TdT) could also play an important role in the detection of ctDNA mutations [76]. A biosensor composed of a triple helix molecular switch (THMS) for recognition, RNase HII, a signal transduction probe (STP), a capture probe fixed on the electrode, and TdT was developed by Wang et al. As target DNA was present, the recognition sequence at THMS was hybridized with target DNA through the complementary pairing principle of Watson–Crick to uncover the STP. Moreover, the hybrid was cut off to release the recognition probe and target DNA for the next recognition cycle in the action of RNase HII. The STP could be captured by CP on the electrode to generate a tree-like DNA decorated by MB; at the same time, current signals would be obtained in collaboration with deoxythymidine triphosphate, TdT, redox-active beads, and assistant probes. This method also showed a satisfactory detection effect for the mutant type with abundant type content (10 fM, 1000:1). In blood samples from five patients with colorectal cancer, this method could accurately detect the single-strand KRAS G12DM mutation. Notably, the strategy is suitable for the detection of different ctDNA mutation sites only by changing the recognition probe.

Since the outbreak of the novel coronavirus in 2019, isothermal amplification (RPA, LAMP, RCA, and RAA) has been applied widely and has overcome the problem of complex temperature programs in PCR, as it only needs to perform rapid amplification at a constant lower temperature. The CRISPR-Cas system, such as SHERLOCK [77], DETECTR [78], SHINE [79], and ADESSO [80], can perform a tube reaction to quickly detect the novel coronavirus with high sensitivity. Could these methods be applied to ctDNA mutation detection? Indeed, we must also recognize that the detection of tumor-driven mutant genes in ctDNA often faces single-base changes; so, an optimization of sgRNA or crRNA in the CRISPR-Cas system has to be performed.

(3) Detection based on nanomaterials

**Figure 3:**
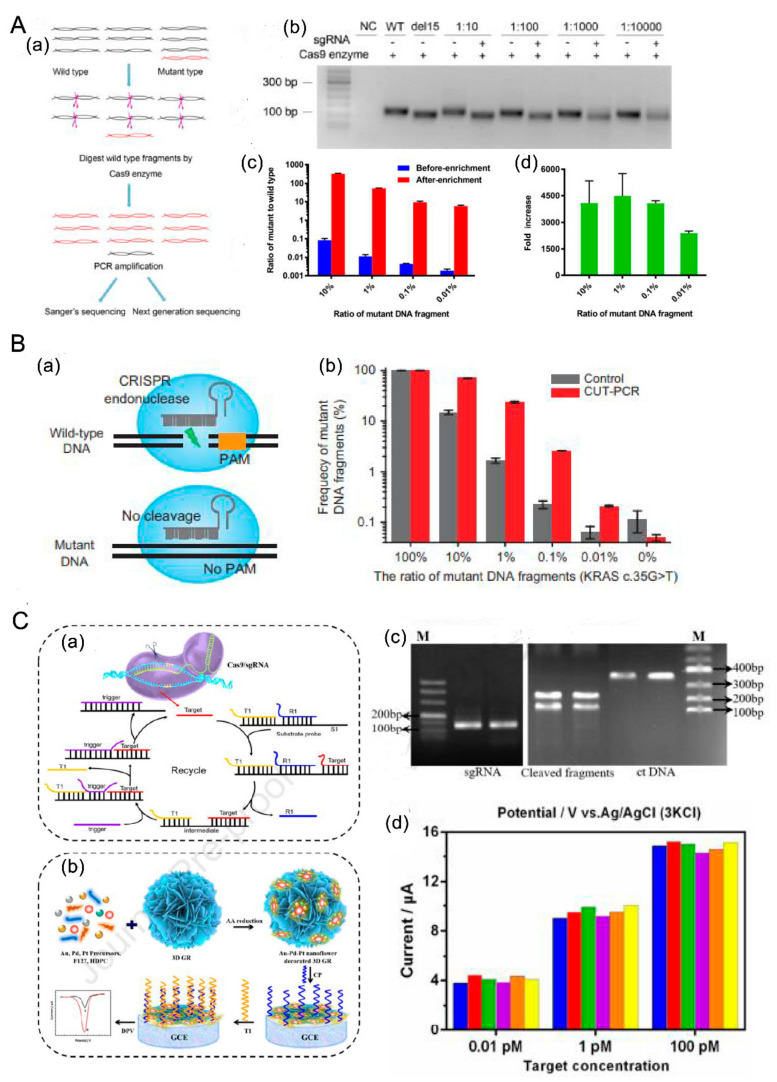

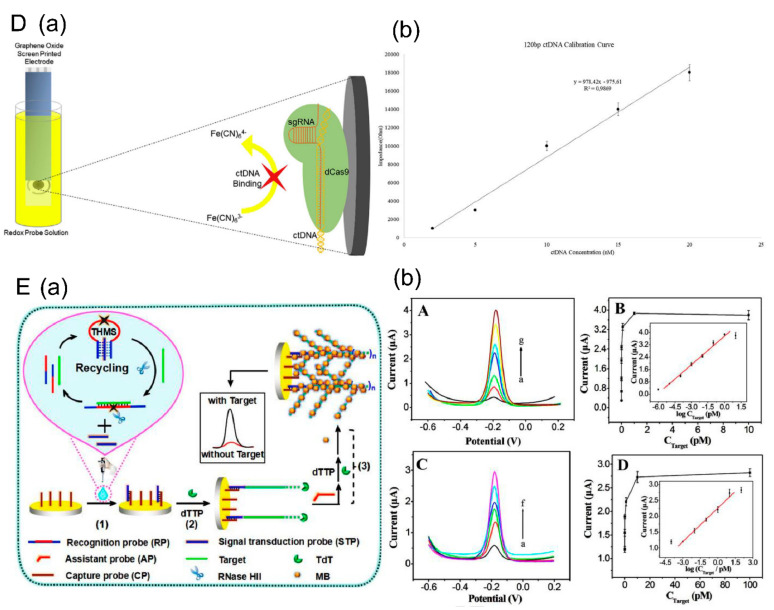

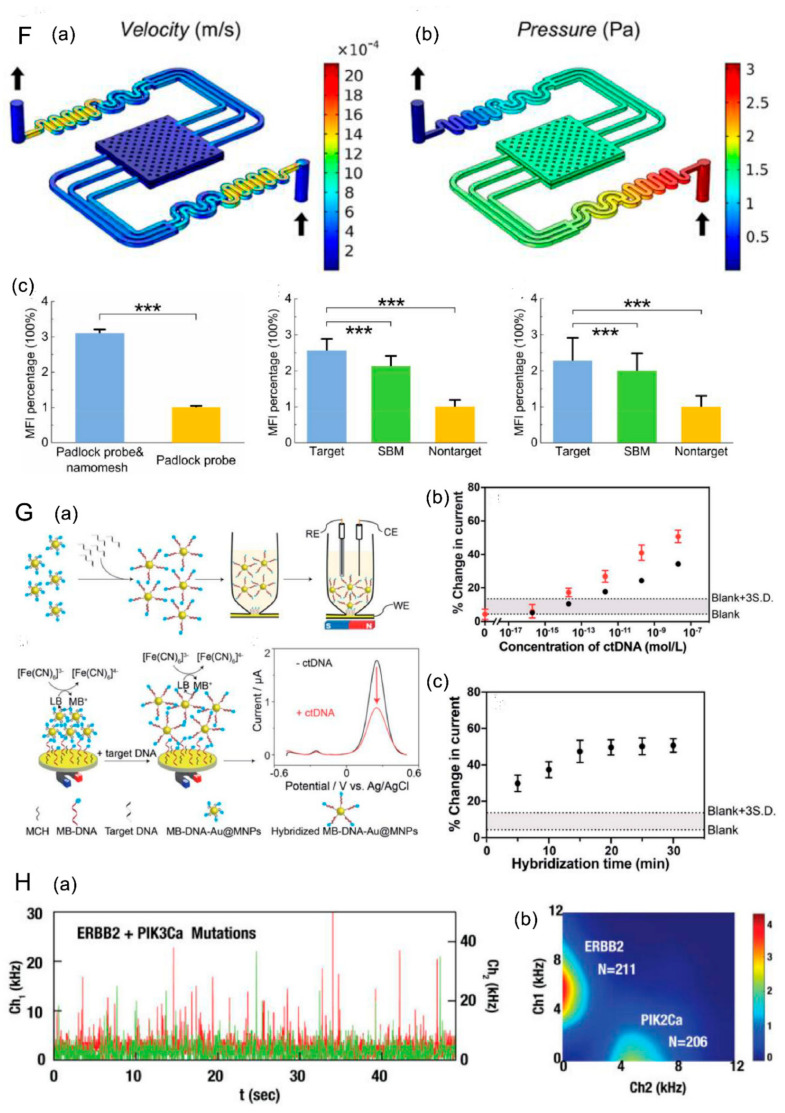
Technologies of ctDNA mutation based on enzyme and nanomaterial. (**A**–**G**) Technology based on CRISPR-Cas system. (**A**) method combining traditional PCR and CRISPR-Cas9 proposed by Wang [71]. (**Aa**) the introduced method mainly includes four steps: Preparation of templates, digestion of WT fragments by the Cas9 enzyme, PCR amplification, and Sanger sequencing or next-generation sequencing. (**Ab**) the PCR results of various template ratios (1/10, 1/100, 1/1000, and 1/10,000), respectively, after enrichment. The templates were mixed using mutant type DNA harboring a 15-bp deletion (c.2235_2249del) and wild-type DNA at various ratios. (**Ac**,**Ad**) the result for EGFR-exon19 15-bp deletion mutant (c.2235_2249del) and the wild-type at different ratios using Cas9/sgRNA digestion plus PCR amplification or without Cas9/sgRNA digestion and the fold increase. (**B**): CUT-PCR [72]. (**Ba**) schematic of the CUT-PCR enrichment process. (**Bb**) for the KRAS (c.35G4T) mutation, targeted deep sequencing after CUT-PCR was treated (red bars) or not (gray bars) were conducted for the plasmid mixtures in which mutant plasmids were originally mixed with wild-type plasmids at a ratio of from 100% to 0.01%. (**C**): Entropy-driven strand displacement reaction [73]. (**Ca**,**Cb**) schematic of the principle of the CRISPR/Cas9-triggered ESDR based on a 3D GR/AuPtPd nanoflower biosensor. (**Cc**) gel electrophoresis of the synthesized sgRNA (left) and DNA samples (right) after Cas9/sgRNA cleavage. (**Cd**) reproducibility of the electrochemical biosensor in different target concentrations (**D**): The combination method of graphene oxide screen printed electrode (GPHOXE) and dCas9 proteins and sgRNA [74]. (**Da**) schematic of CRISPR-dCas9 powered impedimetric biosensor. (**Db**) calibration curve, x-axis represents the ctDNA concentration, y-axis represents the impedance results (ohm). (**E**): A biosensor that was composed of a triple helix molecular switch (THMS) for recognition, ribonuclease HII, signal transduction probe (STP), capture probe fixed on the electrode, and deoxynucleotidyl transferase [76]. (**Ea**) schematic illustration of the dual enzyme assisted multiple amplification electrochemical biosensor. (**Eb**) (**EbA**) DPV responses for the detection of target ctDNA at concentrations of 0, 0.01 fM, 0.1 fM, 1 fM, 0.01 pM, 0.1 pM, and 1 pM (from a to g) with dual enzyme assisted multiple amplification, (**EbB**) Linear relationship between IMB and logarithm of target ctDNA with dual enzyme assisted multiple amplification, (**EbC**) DPV curves for the detection of ctDNA at concentrations of 0, 1 fM, 0.01 pM, 0.1 pM, 1 pM, and 0.01 nM (from a to f) without RNase HII-assisted target recycling amplification, (**EbD**) Linear relationship between IMB and logarithm of target ctDNA without RNase HII-assisted target recycling amplification. Error bars represent standard deviations of three parallel experiments. (**F**): A specific nucleic acid microfluidic capture device based on DNA nanomaterials [81]. Model for the flow simulation in the P-mesh microfluidic capture device. (**Fa**) Velocity. (**Fb**) Pressure. (**Fc**) MFI percentage of Cy5-labeled padlock probe attached on the PVDF membrane. MFI percentage of 1 μM Cy5-labeled ssDNA captured by the P-mesh microfluidic capture device after storage over 6 months. MFI percentage of 1pM Cy5-labeled ssDNA captured by the P-mesh microfluidic capture device after storage over 6 months. Differences between the two groups of samples were tested by *t* test. The level of significance was *** < 0.001. (**G**): A ctDNA ultrasensitive detection method dependent on the targeted recognition of the modified DNA probe on the gold-coated nanomaterials and the target fragment [82]. (**Ga**) Schematic illustration of DNA-mediated reduction of potassium ferricyanide (K3[Fe(CN)6]) by methylene blue (MB). (**Gb**) hybridization-induced change in the SWVs after exposing the sensor to different concentrations of complementary ctDNA target (101 nucleotides) wherein the probe DNA hybridized to the 3′ end (red data points) and the middle (black data points) of the ctDNA. (**Gc**) effect of hybridization time after exposing the sensor to 20 nM complementary ctDNA target (101 nucleotides) on the SWV current change. (**H**): A multiplexed ligation ctDNA single nucleotide nanopore detection method [83]. (**Ha**,**Hb**). the mutated sample exhibited clearly distinguishable optical spikes both in the red and green channels corresponding to passages of the target DNA molecules through the nanopore.

Science has also contributed the ever-changing materials to ctDNA detection. Mei et al. [73] constructed a 3D graphene/Au–Pt–Pd nanoflower biosensor based on a CRISPR-Cas9-targeted coupled entropy-driven strand displacement reaction (ESDR) system for the detection of ctDNA content in patients. This method combines gene editing technology with nanometals. After the ctDNA fragment is targeted by CRISPR-Cas9, specific primers are provided for an entropy-driven strand displacement reaction to generate a triple subunit probe, containing T1, R1, and S1. A complete 3D graphene/Au–Pt–Pd nanoflower is assembled to capture the T1 sequence and output the signal, realizing ctDNA detection. Moreover, the method was successfully applied for the detection of EGFR mutations in ctDNA, with a detection limit of 0.13 pM. Although the detection results were slightly decreased with the serum background, the recovery values were 91.75%–111.5% (RSD: 3.65%–9.26%). This method still has the potential to detect extremely low-frequency ctDNA mutations in complex blood components, and it is expected to be used for clinical practical detection. To detect the DNA content, many researchers have explored methods for constructing DNA nanomaterials from the molecular DNA structure. However, most of them relied on the hydrogen bond in the base complementary pairing principle rather than on the more stable covalent bond, which is easily affected by the Mg concentration or temperature, leading to assembly failure of nanomaterials. Huang et al. constructed a specific nucleic acid microfluidic capture device based on DNA nanomaterials. This device was assembled by the combination of a P-mesh, a PVDF membrane, and a PDMS microchannel layer. The P-nanomesh was formed by a padlock probe corresponding to the target sequence and nanomesh. This method realized the capture of six different target sequences only by changing the targeted probe. The detection efficiency was up to 1 pm, and the single base change sequence could be effectively detected. As to the advantages of high specificity and sensitivity, this sensitive nucleic acid microfluidic capture device is expected to be used for the detection and enrichment of multiple targets in ctDNA, which is further conducive to the diagnosis of patients and formulation of treatment strategies [81]. A ctDNA ultrasensitive detection method was proposed by Chen et al. [82], which mainly depended on the targeted recognition of the modified DNA probe on the gold-coated nanomaterials and target fragment. After centrifugation, washing, and magnet collection, the electrochemical signals of the hybrid products were captured to achieve detection. This method can detect 20 nM to 2 aM DNA fragments, and the minimum detection is 3.3 aM. For practical applications, the detection of sequences with different fragment sizes and the detection of 101 nucleotide ctDNA could also reach 200 pM. However, this method is not sensitive for the detection of single-point DNA mutations, which requires further optimization of the probes and systems. Nanopores are single-molecule sensitive devices for the analysis of nucleic acid biomarkers. Based on this principle, a single nucleotide nanopore detection method, a multiplexed ligation ctDNA, was designed [83]. First, two probes with corresponding fluorescent markers were designed for the mutation site. When the probe and mutation gene were completely annealed and complementary, the ligase could connect the two probes to emit fluorescence signals. The captured fragments were purified using magnetic beads coated with streptomycin, but only the products derived after the connection were separated, and the products were released from the magnetic beads after heating. Finally, an electro-optical nanopore biosensor was utilized to analyze the products and to verify whether there was a targeted mutation in the sample. Using the ERBB2 S310F and PIK3CA H3140R mutations in breast cancer as examples, this method was validated in a synthetic plasmid template and xenograft mouse blood samples, with a sensitivity of 96.6%. Although this method bypasses the PCR and library preparation steps required by traditional sequencing, thus saving time and costs, the operation process is more complex and requires professionals as it cannot be fully automated. The operational steps in this method will need to be simplified further for ctDNA detection in a clinical setting (Figure 3).

(4) Developed Kit

In recent years, non-invasive liquid biopsy technology has been favored by patients. The tumor early-screening market has emerged and continues to grow. Many companies are developing kits for ctDNA detection. We summarized the relevant, currently reported kits (Table 2).

### 4.2. Methylation Detection of ctDNA

There are many detection methods for methylation. Huang and Wang [84] reviewed technologies and bioinformatic approaches for cfDNA methylation analysis, including methyl-sensitive cut counting (MSCC) and HpaII-tiny fragment enrichment by ligation-mediated PCR (HELP) based on methylation restriction enzymes, whole-genome bisulfite sequencing (WGBS), reduced-representation bisulfite sequencing (RRBS), methylated CpG tandems amplification and sequencing (MCTA-seq), targeted bisulfite sequencing, methylation-specific PCR (MSP) based on bisulfite conversion, methylated DNA immunoprecipitation sequencing (MeDIP-seq), methyl-CpG binding domain protein capture sequencing (MBD-seq) based on enrichment, 5hmC-Seal, hmC-CATCH, hydroxy methylated DNA immunoprecipitation sequencing (hMeDIP-seq), and oxidative bisulfite conversion based on 5-hydroxymethylation profiling.

These technologies are generally adopted in the methylation analysis of ctDNA. WGBS was applied to detect the methylation status of ctDNA in the cerebrospinal fluid of patients with medulloblastoma, which is a brain tumor with a high incidence in children and very low frequency of gene mutations, but obvious epigenetic characteristics. The results showed that most of the DNA in the cerebrospinal fluid was ctDNA. Further, the methylation level of the ctDNA methylation island was highly correlated between tumor tissue samples and cerebrospinal fluid, relatively conservative, and highly consistent among individuals, suggesting that the methylation status could be regarded as a biomarker and classification basis for medulloblastoma with high sensitivity and reliability. In addition, a multivariate Cox regression model was established using a training set to verify that the DNA methylation status of characteristic CpG sites in the cerebrospinal fluid of patients with medulloblastoma might be considered a potential prognostic marker for predicting the clinical outcome [85]. The combination of gene mutations and methylation in blood ctDNA detected by WGS and WGBS could specifically target patients with castration-resistant neuroendocrine prostate cancer, which was usually detected by an invasive tissue biopsy and for which it was difficult to obtain complete information due to its heterogeneity [86].

As materials science, optics, and other disciplines have progressed, more advantageous methylation detection methods have been gradually developed. We summarized methylation detection techniques newly developed over the past 5 years.

#### 4.2.1. Discrimination of Rare EpiAlleles by Melt (DREAMing)

DREAMing, which was developed by Thomas et al., achieved single copy level detection for methylation variants [87]. Detailed steps are as follows: first, bisulfite treatment is conducted for ctDNA extracted from blood. Subsequently, the BST sample is diluted to a single BST epiallele in each reaction system, called quasi-digitalization. Next, the diluted sample is amplified by PCR, and the melting curve is monitored in real-time. Finally, the melting curve is analyzed to plot a “DREAM analysis” histogram.

This method combines two concepts, absolute dilution and a melting curve, and realizes the detection of methylation variants without methylation sequencing. This method is simple, cost-effective, and has high sensitivity and specificity. However, this method only detects known loci, and non-specific hybridization caused by amplification products is also an issue that should be considered. The promoter methylation of ctDNA p14ARF and BRCA1 in patients with NSCLC was accurately detected by DREAMing [87]. DREAMing has higher sensitivity and specificity than qMSP, and the methylation density determined by pyrosequencing was positively correlated with the melting temperature of methylation in DREAMing.

#### 4.2.2. DISMIR

DISMIR is a method based on ultra-low-depth WGBS data. Li et al. [88] attributed four steps to the method. First, the tumor-specific methylation region is verified by comparing the blood methylation profiles of patients with and without tumors through ultra-low methylation sequencing. Second, reads corresponding to the specific methylation sites in blood samples of patients with tumors are screened. Third, a deep learning model is constructed by integrating DNA sequences and methylation information, and the d-score parameter is calculated to evaluate the ability to read from cancer tissues. Finally, all d-scores are used to estimate the tumor source score of a plasma sample and to infer whether the patient has cancer. Because this method only needs ultra-low-depth methylation sequencing, the detection cost is relatively low. The establishment of this method depends on the distribution of methylation in the whole genome, rather than the methylation of a single site. Thus, it is designed to be used as a model for methylation as a tumor biomarker. In the early stages, a large number of tumor-specific methylation regions are screened out in the training set, and the corresponding d-score is determined. After the determination of clinical samples by the validation set, it can be used as another method for the clinical diagnosis of patients with a tumor. Considering patients with liver cancer as an example, DISMIR has high sensitivity and robustness.

#### 4.2.3. Detection Based on Digital Microfluidics (dmf)

A method based on pyrosequencing and DMF was used to detect methylation sites in ctDNA. Before pyrosequencing, the samples need to be converted by bisulfite to distinguish methylation sites from non-methylation sites. PCR primers with biotin markers are designed to amplify the template by PCR. The PCR product interacts with streptavidin M280 magnetic beads, and then pyrophosphate sequencing is performed on the MF chip. The chemiluminescence signal emitted by the specific combination of the methylation sequence and sequencing primers is used as the output for the methylation results. Compared with qPCR, sequencing, and mass spectrometry, this method has the advantages of small equipment, short detection time, low detection cost (3.5 USD per run), a low detection line, and high sensitivity reaching 95%. This method could accurately detect 10 pg methylation templates within 30 min [89].

#### 4.2.4. Dual-Recognition-Based Determination

**Figure 4:**
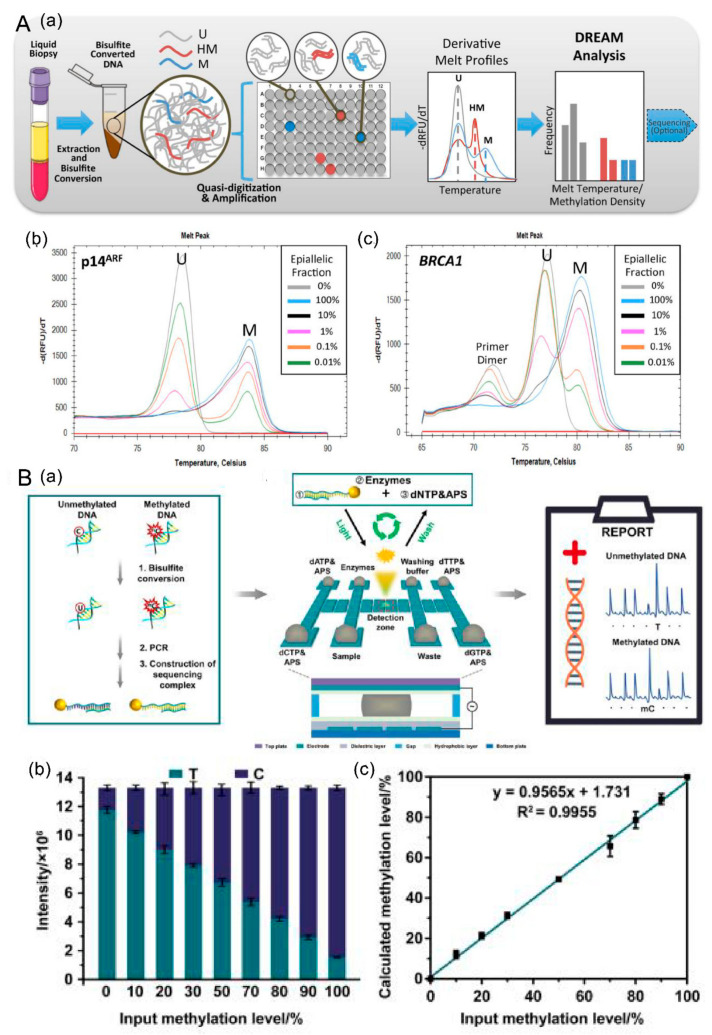

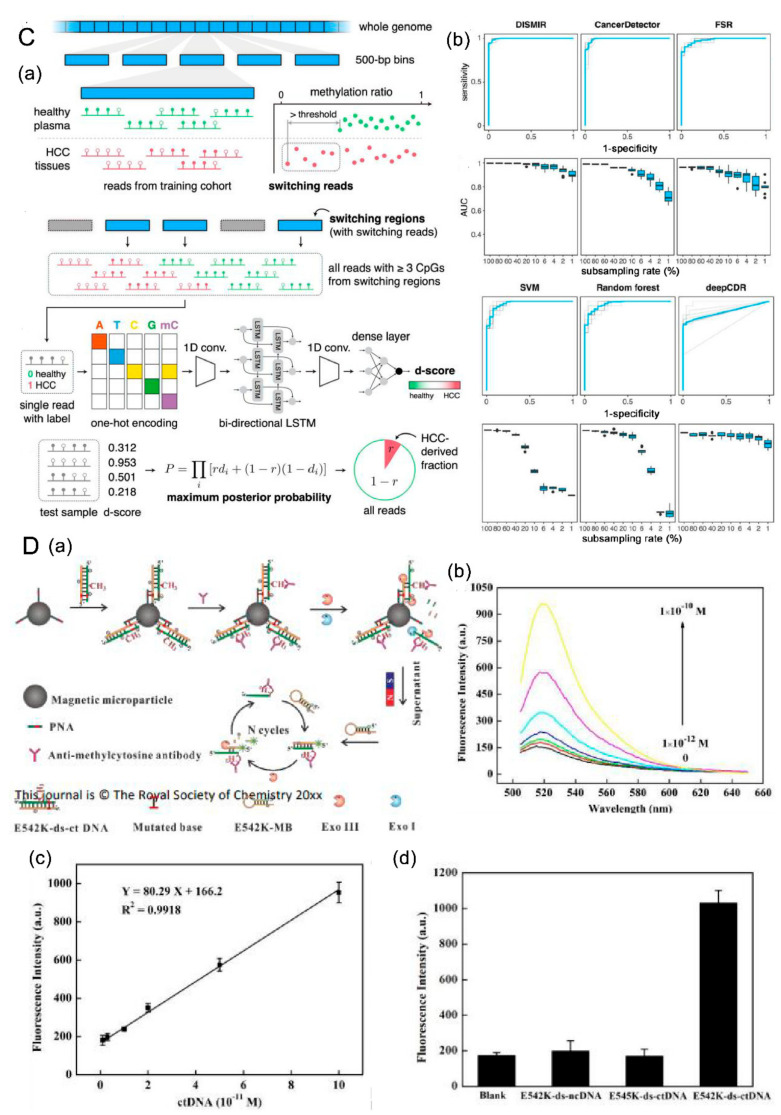
Novel ctDNA methylation technologies. (**A**): Discrimination of Rare EpiAlleles by Melt developed by Thomas. et al. [87]. (**Aa**) DREAMing: analysis of epigenetic heterogeneity at single-copy sensitivity and single-CpG-site resolution. DNA is extracted from a liquid biopsy and undergoes bisulfite treatment (BST). (**Ab**,**Ac**) DREAMing primers optimized for high sensitivity. (**Ab**) for the p14ARF locus at various genomic DNA methylated total epiallelic fractions. (**Ac**) for the BRCA1 locus at various genomic DNA methylated total epiallelic fractions. Both assays exhibit sensitivities that provide detection of epiallelic fractions of 0.01% or lower. (**B**): A method based on pyrosequencing and digital microfluidics [89]. (**Ba**) schematic representation of the DNA methylation analysis based on DMF. (**Bb**) histogram of the signal intensities of T/C at various methylation levels for the first methylation site. (**Bc**) Linear relationship for various levels of input methylation levels for the first methylation site. Data are presented as mean ± SD from triplicate samples. (**C**): DISMIR based on ultra-low-depth WGBS data [88]. (**Ca**) Overview of DISMIR. (**Cb**) results of DISMIR and other methods on HCC diagnosis. (**D**): Dual-recognition-based determination grounded on peptide nucleic acid (PNA) and terminal protection of small-molecule-linked DNA (TPSMLD) [90]. (**Da**) the mechanism of the dual-recognition fluorescence biosensor for E542K-ds-ctDNA. (**Db**) fluorescence spectra of the dual-recognition fluorescence biosensor upon the addition of increasing concentration of E542K-dsctDNA. (**Dc**) calibration curve for E542K-ds-ctDNA detection. (**Dd**) bar chart of the fluorescent intensities in the presence of different DNA sequences.

Dual-recognition-based determination of ctDNA was established based on peptide nucleic acid (PNA) and terminal protection of small-molecule-linked DNA (TPSMLD). PNA is a versatile probe functioning to detect single base pair gene variants. TPSMLD could be used to protect the terminal groups of small-molecule DNA fragments, but avoid ExoI digestion when binding to the target protein. Based on these concepts, Chen et al. [90] developed a method for the dual detection of ctDNA. The term refers to both mutation and methylation detection. PNA complementarily paired with the target sequence was designed to realize the specific detection of mutations since the melting temperature difference between PNA and the mutation sequence and the normal sequence was 11 °C. In addition, the successful detection of methylation depended on specific recognition of anti-5-MC and methylation sites. This method was successfully applied to detect the mutation of PIK3CA E542K in double-strand ctDNA and in clinical serum samples, with high specificity and sensitivity and a low detection limit of 0.3161 pM. This method can be used when mutation and methylation occur simultaneously, but it has the disadvantage of low sensitivity (Figure 4).

## 5. Clinical Application of ctDNA during Cancer Progression

### 5.1. ctDNA and Early Diagnosis of Tumor

It is well-known that cancer is the consequence of multiple gene mutations, which seriously threaten the health of patients. Early detection and diagnosis are currently major factors for the success of tumor treatment and for effectively improving the quality of life of patients. At present, the early diagnosis of tumors is mainly dependent on gene detection, and ctDNA released by pathological tumors into the blood has unique and excellent advantages. The specificity and sensitivity of molecules, such as ctDNA, cfDNA, CTC, and multiple biomarkers, were compared in existing liquid biopsies for the early diagnosis of cancer [44]. Unexpectedly, the detection efficiency of cfDNA and ctDNA was better than that of other parameters. Luo et al. [41] constructed a diagnostic score (cd-score), which composed of nine methylation markers in the ctDNA of patients with colorectal cancer. The cd-score had excellent sensitivity and specificity for the diagnosis of colorectal cancer, which were superior to those of the conventional colorectal cancer diagnostic marker CEA (AUC: 0.96 vs. 0.67). Moreover, the cd-score was also of use in the response to colorectal cancer tumor staging, treatment methods, and the identification of minimal residual disease after treatment. In addition, in a prospective study involving 16,890 participants, the methylation marker cg10673833 was selected and identified as an early diagnostic marker for high-risk patients with colorectal cancer, with high specificity (86.8%) and sensitivity (89.7%). Leung et al. [91] detected the highest frequency of EGFR, KRAS, and TP53 mutations in the ctDNA of 166 patients with lung cancer. In terms of cancer diagnosis, compared with conventional clinicopathological results, the ctDNA diagnosis had a 98% positive predictive ability, 89% specificity, and 85% sensitivity, but the negative predictive value was 35%, which is a serious and urgent problem that should be solved. It has been reported that pulmonary nodules are related to the formation of lung cancer lesions. Liang, in collaboration with 23 medical centers in China, collected ctDNA from 10,560 patients for next-generation sequencing and observed that pulmonary nodules can be diagnosed according to the methylation of ctDNA in patients [92]. KRAS is the most frequently mutated gene in pancreatic cancer, and the most common mutation sites are c.35 > A, p.G12D and c.38G > A, p.G13D. It was found that the KRAS mutant allele fraction (MAF) in ctDNA plays an important role in the diagnosis of pancreatic cancer. The combination of KRAS MAF with the conventional biomarker CA19-9 can significantly improve detection sensitivity, reaching 82%. Moreover, in the same patient, the KRAS MAF in ctDNA was consistent with that in the lesion tissue [93]. In addition, ctDNA could be combined with ultrasonic elastography to enhance the assessment of changes in the stiffness of breast cancer lesions and to realize the early diagnosis and prognosis of breast cancer [94].

Moreover, Cohen et al. describe a blood test, named CancerSEEK for cancers of the ovary, liver, stomach, pancreas, esophagus, colorectum, lung, or breast. The test contains 61 mutation sites of 16 common mutant genes, such as PIK3CA, APC, EGFR, TP53, and KRAS, and 41 cancer-specific protein biomarkers, such as AFP, CA-125, sHER2/sEGFR2/sErbB2, etc. The mutation is mainly used to analyze whether there is cancer to improve the detection sensitivity, and the tumor-specific protein biomarkers can be used for cancer types, improving the specificity of detection (>99%). In 1005 patients with the above eight tumors, the median positive rate of this method was 70% and the false positive rate was low (7/812). However, as the test is only compared to normal healthy people, will the positive rate increase when the method is applied to sub-healthy people? How to solve this practical problem requires researchers to further decode [95]. Similarly, Lennon et al. named DETECT-A (Detecting cancers Earlier Through Elective mutation-based blood Collection and Testing), combining blood tests and full-body diagnostic positron emission tomography-computed tomography (PET-CT). Blood tests also detect tumor-specific mutations, followed by PET-CT for re-diagnosis, localization, and precise treatment to improve treatment. A total of 134 subjects were screened from 9911 volunteers by the use of blood tests. Among 134 positive patients, 64 of them showed tumor-related imaging information by PET-CT and then confirmed by biopsy technology and 26 patients had cancer and subsequently received corresponding clinical treatment. Although it is complex, DETECT-A is accurate and reduces over-diagnosis and over-treatment decision making that could be included in routine clinical care [96]. For early tumor screening, methylation can be an independent predictor. Chen and his colleagues developed a ctDNA methylation detection scheme called PanSeer by screening 477 tumor-specific, 10613 CpG sites from some well-known tumor-related genes and sequencing blood samples using genome-wide bisulfite sequencing (WGBS). The method was used to test healthy subjects in the Taizhou Longitudinal Study for four years, and 191 of the 605 asymptomatic individuals were diagnosed with stomach, esophageal, colorectal, lung, and liver cancers. PanSeer detection detects cancer by targeting a limited number of genomic regions with aberrant methylation common in different cancer types, but it is likely not to predict patients who will develop cancer in the future. PanSeer is most likely to identify asymptomatic patients who have cancerous growths but cannot be detected by current detection methods as early as possible [97]. In the largest clinical genomics program, methylation detection in cfDNA still showed excellent tumor early-screening performance, and more than 50 cancer types were successfully detected at different stages with a specificity of 99.3% [98]. Nasopharyngeal carcinoma (NPC) is a type of cancer with unique geographical characteristics. It is highly prevalent in the southeast and south of China, and the occurrence and development of cancer are closely related to the Epstein–Barr virus (EBV) infection. Almost every cancer cell contains the EBV genome. Therefore, it is generally accepted that circulating tumor EBV DNA (ctEBV DNA) can be used as a unique biomarker for NPC [99]. Lam et al. screened 20,174 asymptomatic individuals for NPC by target-capture sequencing. The results showed that compared with non-NPC subjects, the plasma ctEBV DNA content of NPC patients increased and the fragment length was significantly prolonged, suggesting that the detection of ctEBV DNA provides a favorable tool for NPC screening [100]. In addition, ctEBV DNA also plays an important clinical role in the prognosis of NPC [101], risk stratification [102], and recurrence monitoring [103].

### 5.2. ctDNA and Prognosis of Tumor

ctDNA is an excellent tumor prognostic marker. In FIRSTANA and PROSELICA, two prospective phase three clinical trials, ctDNA was sequenced by low-pass whole-genome sequencing. Univariate analysis and a stratified multivariate analysis showed that the ctDNA score was associated with the overall prognosis of prostate cancer [104]. A high level of ctDNA was detected by Amanda et al. [105] in 24 patients with positive brain metastases of breast cancer using ULP-WGS, but none of the patients with negative ctDNA were detected. Further, the diagnostic effect of ctDNA was far better than that of the current gold standard, namely, cerebrospinal fluid cytology and the conventional diagnostic method MRI. Moreover, as patients received intrathecal treatment, the decrease in ctDNA content was related to their prolonged survival. Continuous detection of ctDNA in the cerebrospinal fluid of patients could predict disease progression after intrathecal treatment. For patients with metastatic bladder cancer, the alteration of the FGFR3 gene in ctDNA was related to increased sensitivity to erdafitinib and prolonged progression-free survival of patients [24]. BRCA1, SLFN11, and USP44 methylation markers were screened by real-time quantitative methylation PCR in cancer tissues and peripheral blood of patients with high-grade serous ovarian cancer. The methylation level of SLFN44 in the ctDNA of patients with advanced cancer was significantly correlated with poor progression-free survival [106]. A meta-analysis [107] of eight studies involving 672 patients with ovarian cancer showed that ctDNA was associated with the tumor size and stage. In addition, a higher level of ctDNA was associated with poor prognosis. Thus, ctDNA can be regarded as a potential molecular prognostic indicator for patients with ovarian cancer. The molecular tumor burden index (mTBI) is a term related to tumor progression. Compared with patients with mTBI > 0.02% in the initial tumor assessment of breast cancer, patients with breast cancer with an mTBI < 0.02% have better progression-free survival and overall survival. The level of mTBI in ctDNA showed a significant downward trend before clinical observation or imaging detected that the tumor volume had decreased, suggesting that mTBI can be used as an effective prognostic marker for breast cancer, which helps to identify patients with good therapeutic effects and to further optimize their targeted therapy [108]. In addition to solid tumors, ctDNA still plays an important role in neuroendocrine neoplasms [109]. In neuroendocrine tumors, the existence of ctDNA was related to the grade and location of primary tumors. Compared with ctDNA-negative patients, ctDNA-positive patients showed shorter overall survival and a higher risk of death, contributing to the prediction of tumor progression. Can ctDNA be used for targeted drug selection and therapeutic effect detection in neuroendocrine neoplasms? This is a question worthy of further exploration with extremely meaningful, innovative direction. Targeting tumor fraction (TF) in plasma ctDNA as an indicator, univariate/multivariate analysis was performed in 1725 cancer patients (198 Metastatic Castration-Resistant Prostate Cancer, 223 metastatic colorectal cancer, 902 NSCLC, and 402 breast cancer). The results showed that the overall survival of patients was independently and consistently correlated with a TF > 10%, enabling the accurate grading of cancer treatment and reducing the possibility of over-treatment [110]. Mo et al. [111] selected 191 meaningful methylated haplotype markers from 11,878 CpG sites and constructed algorithms in advanced adenoma and CRC training sets. In the blind verification set, the AUC of advanced adenoma and CRC was 0.903 and 0.937, respectively. Compared with patients with low methylation levels, patients with high preoperative methylation levels have a worse prognosis.

### 5.3. ctDNA and Tumor Molecular Subtyping Profiles

Cancers are usually complex diseases involving multiple genes. Different stages have different gene expression profiles. The heritability, individual differences, and complexity of cancer’s molecular mechanisms vary. In addition, cancer can be divided into different subtypes according to its molecular characteristics. The most common is based on the human epidermal growth factor receptor (HER) oncogene. Based on the Ki-67 labeling index, which is used to detect cell proliferation and analyze the status of the estrogen (E) and progesterone receptor (PgR), breast cancer is divided into basal-like, luminal A, and luminal B subtypes [112]. Accurate treatment strategies for different cancer subtypes are urgently needed, including accurate diagnosis, prognostic stratification, tumor staging, recurrence monitoring, and drug development. These steps can improve the efficiency of treatment, save cancer patients’ lives, and improve their quality of life. Recently, it has been found that ctDNA can also play a role in the molecular typing of cancers. Shi et al. [113] extracted ctDNA from more than 5000 Chinese lung cancer patients and found that ctDNA and bTMB levels were significantly lower in non-small cell lung cancer patients than in small cell lung cancer patients (p < 0.001), regardless of the cancer being adenocarcinoma, squamous carcinoma, adenosquamous carcinoma, or large cell lung cancer. Recent studies have reported that the subtypes of small cell lung cancer contained atonal bHLH transcription factor 1 (ATOH1), pOU class 2 homeobox 3 (POU2F3), neurogenic differentiation factor 1 (NEUROD1), and achaete–scute complex homolog-like (ASCL1) [114,115]. Additionally, NEUROD1, ASCL1HE, and double negative subtypes were the dominant subtypes according to the analysis of 177 SCLC clinical samples [116]. In this study, 366 differential methylation regions (DMRs) were screened using the methylation sequencing profile of 59 cell lines from the study of Francesca et al. [117], and three major subtypes of SCLC were successfully distinguished in fifty-six SCLC clinical blood samples by efficient DMRs, i.e., 73%, 13%, and 14%, and verified by immunohistochemical results. For the majority of de novo metastatic castration-sensitive prostate cancer (mCSPC) patients, tumor tissue and cfDNA sequencing alone were not sufficient to provide somatic information about the patient. The combination of ctDNA and tissue revealed gene-level changes in mCSPC patients, such as extensive TP53 mutation and MSH2 truncating mutation. Therefore, it is a favorable method for evaluating tumor molecular subtypes, laying a solid foundation for the development of the next targeted treatment strategies for cancer patients [118]. Moreover, Gao [119] optimized a WGBS-based ctDNA methylation detection method and identified 15 methylated biomarkers (DMRs) that could significantly distinguish between early and advanced breast cancer patients and healthy volunteers (AUC: 0.996). In addition, 12 ctDNA DMRs were identified as potential biomarkers for discriminating clinical subtypes of breast cancer, which were validated in the training set of 38 breast cancer patients and the validation set of 123 patients. The 12 biomarkers effectively distinguished ER (+) and ER (−) breast cancer patients (AUC values were 0.984 and 0.780, sensitivity was 93% and 73%, and specificity was 93% and 87%, respectively). This method was also applicable to hepatocellular carcinoma and lung cancer and could effectively differentiate their molecular subtypes. In summary, ctDNA can also reveal the molecular markers associated with tumor typing and contribute to tumor typing.

### 5.4. ctDNA and Tumor Recurrence Monitoring

ctDNA plays an important role in the monitoring of tumor recurrence. For instance, ctDNA mutations were detected in nine patients with gastric cancer after surgery, of which six patients had cancer recurrence and died of complications caused by cancer metastases. On the contrary, 11 patients without a ctDNA mutation had no recurrence after surgery and had a good quality of life, suggesting that ctDNA has the potential to become a detection parameter for postoperative prognosis and recurrence in patients with gastric cancer [120]. Qiu et al. [121] constructed a combined model of longitudinal ctDNA analysis and time-to-recurrence. Compared with the traditional Cox method, the combined model had a better prediction potential for the recurrence of NSCLC. With the extension of follow-up time, the ctDNA level of patient P062 remained unchanged. The recurrence rate was low, and the condition was stable. On the contrary, the ctDNA level of patient P017 gradually increased. The model predicted that the recurrence risk was significantly increased, and the recurrence occurred shortly after the last follow-up, indicating that the model could realize the dynamic monitoring of the ctDNA level in patients with NSCLC and predict the recurrence risk. According to the prediction results, neoadjuvant chemotherapy was used in advance to improve the prognosis of patients. After a resection of hepatocellular carcinoma, the positive rate of ctDNA was significantly decreased. Patients with major pathological response (MPR) or complete pathological resection had a lower ctDNA content than patients with non-MPR. After neoadjuvant therapy, the positive rate of ctDNA in patients with MPR increased from 33.3% to 83.3%, and the ctDNA in patients with non-MPR also showed a rising trend. The correlation between this rising trend and cancer recurrence was accurately verified in several confirmed patients, which indicates that ctDNA might play a role in evaluating the clinical pathological response and tumor recurrence [122]. Although previous research has found that changes in blood ctDNA in patients with cancer are associated with disease progression, sequencing of the diseased tissues is still needed to align ctDNA mutations with the original mutation of the tumor and to ensure the effectiveness of the detection. However, for some patients, it is not easy to obtain tissue samples, and the mutation types in tissue samples obtained by surgeons may be quite different from those in ctDNA because of tumor heterogeneity. The integration of ctDNA genomics and epigenetics in patients with colorectal cancer, as demonstrated by Parikh et al. [123], could be used for the evaluation of recurrence and prognosis of patients using plasma-only samples and for the realization of longitudinal disease monitoring. Total neoadjuvant therapy is an effective treatment for patients with locally advanced rectal cancer. In the GEMCAD 1402 multicenter clinical trial, Joana et al. conducted regular follow-ups of 180 patients with rectal cancer before and after surgery. The results showed that the preoperative ctDNA level could predict the response of patients to total neoadjuvant therapy and the recurrence and survival of patients. In addition, nine patients with preoperative ctDNA had disease metastases, seven had single organ metastases, and two had multiple organ metastases. The sensitivity and specificity of preoperative ctDNA for liver metastases were high (75% and 90%, respectively). However, it was difficult to detect ctDNA before an operation in patients with lung and peritoneal recurrence, indicating that ctDNA is related to the recurrence site of patients. This conclusion needs to be further verified in larger clinical samples. In colorectal cancer, being ctDNA-positive indicated that the effect of tumor recurrence was obvious. A total of 125 patients received ctDNA detection before surgery, and 122 patients were ctDNA-positive. After surgery, 14 of the 16 patients with recurrence were ctDNA-positive. After receiving surgery for 30 days, the recurrence rate of ctDNA-positive patients was significantly higher than that of ctDNA-negative patients. Not only surgical resection showed this phenomenon, but also patients who received neoadjuvant chemotherapy. All seven ctDNA-positive patients relapsed after neoadjuvant chemotherapy [124]. A small amount of residual tumor cells in the body is called minimal residual disease (MRD). In Loupakis‘ paper, ctDNA-based MRD was significantly negatively correlated with disease-free survival in patients with metastatic colorectal cancer (p < 0.001), suggesting it could be a prognostic factor for metastatic colorectal cancer [125]. In addition, ctDNA combined with the biomarker CEA also has a good prognostic effect [126]. In addition, the role of ctDNA in MRD and predicting the recurrence of melanoma [127,128], lung cancer [129], localized colon cancer [130], pancreatic ductal adenocarcinoma [131], and other cancers have been gradually confirmed.

### 5.5. ctDNA and Clinical Medication Guidance

Drug therapy, whether chemotherapy or immunotherapy, is an individualized choice. After a period of drug treatment, some patients will develop drug resistance. Through ctDNA detection, patients can receive individualized medication and choose more favorable drugs for treatment. The therapeutic effect of adjuvant chemotherapy on patients with stage III colon cancer is obvious, but the clinical benefits of adjuvant chemotherapy for patients with stage II cancer with ineffective surgical treatment are not clear. A randomized trial, called the circulating tumor DNA Analysis Informing adjuvant Chemotherapy in Stage II Colon Cancer (DYNAMIC), was designed to evaluate whether a method guided by ctDNA could reduce the frequency of adjuvant treatment use without compromising the risk of recurrence [132]. The trial, including 302 patients for ctDNA-guided management and 153 patients for standard management, showed that ctDNA detection could reduce the use of adjuvant therapy for patients with stage II colon cancer, when compared to standard management, and did not imperil recurrence-free survival. Therefore, ctDNA testing can predict whether patients with stage II colon cancer can benefit from adjuvant chemotherapy, thereby reducing psychological and physiological injuries and unnecessary medical expenses for patients. In an exploratory analysis evaluating the overall survival of patients with HR+/HER2+ advanced breast cancer (ABC) treated with CDK4/6 inhibitors, PALOMA-3, patients without TP53/PIK3CA/ESR2 mutations in ctDNA showed better overall survival and progression-free survival when treated with palbociclib plus fulvestrant, indicating that a driver gene mutation in ctDNA can support the predictive value for clinical medical management [133]. Lu et al. [134] constructed a ctDNA sequencing-based tumor mutation (TMI) model combined with blood tumor mutation burden (bTMB) in ctDNA. The sensitive blood tumor mutation burden (sbTMB) and susceptibility score (UMS) were used to evaluate whether patients with NSCLC can benefit from the anti-angiogenic agent anlotinib, the chemotherapeutic docetaxel, or the immune checkpoint inhibitor atezolizumab. The model included many influencing factors, such as: (a) multi-level gene mutations, (b) clinical characteristics (including the pathological type, driver gene status, number of metastases, sex, and smoking history), and (c) clinical effect comparisons by chemical immunotherapy or immunotherapy between TMI and bTMB. In addition, TMI could effectively predict the response to docetaxel or atezolizumab in patients with NSCLC, which could be considered an ideal biomarker. Moreover, patients with low TMI who received atezolizumab treatment generally showed improved overall survival. According to the principle of precision therapy, accurate drug selection can reduce the wastage of medical resources and avoid the possibility of ineffective treatment for patients. TMI derived from ctDNA can achieve this goal, and this method cannot rigidly adhere to NSCLC. The construction of corresponding TMI models in various cancers can be universal. While an anti-EGFR monoclonal antibody was used to treat patients with RAS wild-type metastatic colorectal cancer, the occurrence of drug-resistant mutations, such as in the RAS, BRAF, and EGFR genes, after a period of treatment would greatly reduce the therapeutic effect in patients, and these mutations could be detected in patients’ peripheral blood. In this regard, an open-label, single-arm phase II clinical trial, named CHRONOS [135], was first proposed to detect resistant mutations in patients receiving the EGFR monoclonal antibody panitumumab, thereby guiding precise clinical medications to obviate toxic and invalid treatments. In the course of panitumumab treatment in 52 patients, ddPCR was used to monitor the ctDNA mutation panel composed of KRAS, BRAF, and EGFR extracellular domain (ECD). There was at least one drug-resistant mutation in 16 patients with poor treatment efficacy, suggesting that the emergence of drug-resistant mutations is related to panitumumab failure. Based on the “zero mutation ctDNA triage” principle proposed by the author, 36 patients with no ctDNA mutations were continuously treated with panitumumab in the trial. The detection of ctDNA could predict the treatment response of patients, and the treatment time provided by ctDNA was more personalized and accurate than that provided in advance. Briefly, the detection of a ctDNA mutation in the CHRONOS trial enables the maximization of the therapeutic effect of panitumumab treatment for patients with metastatic colorectal cancer, avoids adverse reactions with time, and provides more accurate treatment strategies for patients with ctDNA mutations.

In the phase II B-F1RST trial that evaluated whether bTMB could be a novel biomarker for locally advanced or metastatic stage III B–IVB NSCLC treated with atezolizumab, the objective response rate of intent-to-treat patients was altered as the threshold value of bTMB. Finally, the trial report demonstrated that bTMB showed a positive correlation with longer overall survival, indicating that bTMB could be another predictive biomarker for a patient’s immunotherapy [136].

## 6. Discussion and Perspective

ctDNA, derived from tumor cells, reflects a series of changes in the process of tumor development, such as gene mutation, DNA methylation, rearrangement, MSI, LOH, etc. It can be used as an important and favorable marker for tumor management and plays a key role in early tumor screening, early diagnosis, prognostic monitoring, tumor molecular subtyping and clinical drug selection. Current research results show that the use of ctDNA in NSCLC and colorectal cancer may be the reason why the driver genes of these two cancers have been studied more thoroughly than those of other cancer types.

The whole ctDNA management includes four aspects: extraction, detection, analysis, and application. In terms of extraction, at present, ctDNA is derived from the apoptosis of tumor cells, and the actual source is clearly not only peripheral blood, which is the most common source, but also urine, feces, ascites, and cerebrospinal fluid, among others. Different sources of ctDNA require different pre-treatment methods and final extraction methods. The most commonly used kit is Qiagen’s QIAamp Circulating Nucleic Acid Kit. Detection is the most important part of ctDNA management. To date, researchers have developed many detection methods for the most common mutations and methylation changes in ctDNA, most of which rely on sequencing methods. However, due to the extremely low content of ctDNA and low proportion of mutations and methylation, only deep sequencing can be used, which leads to a sharp increase in sequencing costs. In addition, many researchers have also developed other detection methods from the perspective of enzyme and materials science. While ensuring high sensitivity and high specificity, the detection process is, at least to some extent, complicated. Further optimization is needed to generate products for clinical testing. Since the global outbreak of the novel coronavirus in 2019, a series of virus detection methods have been developed, such as the CRISPR-Cas system combined with isothermal amplification (including RPA, LAMP, RCA, and RAA). Similarly, the methods for the detection of ctDNA continue to be updated. In addition, with the rapid development of materials science, optics, and other disciplines, SPR, microfluidic technology, surface resonance technology, and graphene adsorption theory can also be applied to ctDNA detection. Can this lead to the development of a new generation of ctDNA detection technology? At present, many in vitro diagnostic companies have launched some ctDNA detection kits, but their detection limit is generally 0.1%. It is necessary to further optimize the detection for lower ctDNA levels to guide the subsequent application of ctDNA in tumor management. Data analysis is generally matched with detection technology. Finally, with regard to the application of ctDNA detection, the most common application is in colorectal cancer and NSCLC, involving early diagnosis, prognostic monitoring, recurrence monitoring, and guiding drug resistance. However, for other solid tumors, such as liver cancer, gastric cancer, and breast cancer, ctDNA detection is still in a stagnation stage and, therefore, has great room for development. Furthermore, there are some practical and fatal problems that need to be considered. These include: a) ctDNA is less detached from the tumor and has a low percentage in cfDNA; b) it is less likely to reproduce tumor heterogeneity in ctDNA; c) mutations in ctDNA are not specific to particular cancers and are also highly mutated in other cancer types. For example, PIK3CA E545K / E542K is the second most recurrent PIK3CA mutation in breast cancer, but it also occurs highly in colon adenocarcinoma, lung adenocarcinoma, and bladder urothelial carcinoma. Nothing is perfect. Combining ctDNA with other specific tumor markers and detection methods may be an unexpected choice.

ctDNA is an ideal tumor marker which reflects the change in the tumor gene level. Once the mystery of ctDNA is solved, researchers can design corresponding targeted inhibitors, and clinicians can treat the sources of cancer, monitor the real-time therapeutic effects, and evaluate patient prognoses.

## 7. Conclusions

ctDNA is an ideal tumor marker which can effectively reflect the dynamic changes in early tumor screening, diagnosis, tumor molecular subtyping profiles, prognosis, recurrence monitoring, and medication guidance. A series of more sensitive detection methods have been gradually developed and are expected to be put into clinical application as soon as possible.

## Figures and Tables

**Table 1:** Summary of selective kits for circulation tumor DNA extraction.

Origin of ctDNA	Selective Kit
Blood	QIAsymphony DSP Virus/Pathogen Midi Kit
	MagMAX™ Cell-Free DNA Isolation Kit
	DSP Circulating DNA Kit
	QIAamp Circulating Nucleic Acid Kit
	EliteHealth cfDNA Extraction Kit
Urine	QIAamp Circulating Nucleic Acid Kit
Cerebrospinal fluid	QIAamp Circulating Nucleic Acid Kit
	QIAsymphony DSP Virus/Pathogen Midi Kit
	QIAsymphony DSP Circulating DNA Kit
Feces	Fast DNA Stool Mini Kit
Ascites	QIAamp Circulating Nucleic Acid Kit
Pleural effusions	QIAamp Circulating Nucleic Acid Kit

**Table 2:** ctDNA mutation detection kit.

Kit	Target	Cancers	Technology	Specificity	LoD	Company	Country
AVENIO ctDNA Expanded Kit	77 gene panel (including 192Kb, *ABL1*, *AKT1*, *BRAF*, and *PIK3CA*)	Lung cancer and colorectal cancer	Next-generation sequencing	>99%	0.10%	Roche	Switzerland
AVENIO ctDNA Target Kit	17 NCCN guideline-aligned genes (81 kb)	Lung cancer and colorectal cancer	Next-generation sequencing	>99%	0.10%	Roche	Switzerland
AVENIO ctDNA Surveillance Kit	17 NCCN guideline-aligned genes plus 471 frequently mutated, disease-associated regions across 197 genes	Lung cancer and colorectal cancer	Next-generation sequencing	>99%	0.10%	Roche	Switzerland
Super-ARMS^®^ EGFR Mutation Detection Kit	Covers the 42 most frequent *EGFR* mutations	Non-small cell lung cancer	Real-time PCR	100%	0.20%	AmoyDx	China
OncoBEAM EGFR Kit V2 (RUO)	36 *EGFR* mutations of oncogene exons 18, 19, 20, and 21 (including T790M and C797S)	Non-small cell lung cancer	Highly sensitive BEAMing digital PCR technology	>90%	0.01%	Sysmex	Japan
Guardant360^®^ CDx	Single nucleotide variants (SNVs) and insertions and deletions (indels) in 55 genes; copy number amplifications (CNAs) in 2 genes; fusions in 4 genes	Non-small cell lung cancer	Qualitative next-generation sequencing	100%	0.20%	Guardant Health	USA
FoundationOne^®^ Liquid CDx	Substitutions and insertions and deletions (indels) in 311 genes, including rearrangements and copy number losses only in *BRCA1* and *BRCA2*	Non-small cell lung cancer and prostate cancer	Qualitative next-generation sequencing	100%	0.40%	Foundation Medicine	USA
Guardant Reveal™	Not published	Early-stage colorectal, breast, and lung cancers	Next-generation sequencing	100%	0.01%	Guardant Health	USA
